# B7-H3/CD276: An Emerging Cancer Immunotherapy

**DOI:** 10.3389/fimmu.2021.701006

**Published:** 2021-07-19

**Authors:** Wu-Tong Zhou, Wei-Lin Jin

**Affiliations:** ^1^ Institute of Nano Biomedicine and Engineering, Shanghai Engineering Center for Intelligent Diagnosis and Treatment Instrument, Department of Instrument Science and Engineering, Key Laboratory for Thin Film and Microfabrication Technology of Ministry of Education, School of Electronic Information and Electronic Engineering, Shanghai Jiao Tong University, Shanghai, China; ^2^ Institute of Cancer Neuroscience, Medical Frontier Innovation Research Center, The First Hospital of Lanzhou University, The First Clinical Medical College of Lanzhou University, Lanzhou, China

**Keywords:** B7-H3/CD276, immune checkpoint, tumor microenvironment, tumor immunology, cancer immunotherapy

## Abstract

Immunotherapy aiming at suppressing tumor development by relying on modifying or strengthening the immune system prevails among cancer treatments and points out a new direction for cancer therapy. B7 homolog 3 protein (B7-H3, also known as CD276), a newly identified immunoregulatory protein member of the B7 family, is an attractive and promising target for cancer immunotherapy because it is overexpressed in tumor tissues while showing limited expression in normal tissues and participating in tumor microenvironment (TME) shaping and development. Thus far, numerous B7-H3-based immunotherapy strategies have demonstrated potent antitumor activity and acceptable safety profiles in preclinical models. Herein, we present the expression and biological function of B7-H3 in distinct cancer and normal cells, as well as B7-H3-mediated signal pathways in cancer cells and B7-H3-based tumor immunotherapy strategies. This review provides a comprehensive overview that encompasses B7-H3’s role in TME to its potential as a target in cancer immunotherapy.

## Introduction

Immunotherapy that results in remarkable and durable responses across many different tumor types in patients by promoting antitumor immune responses has revolutionized the treatment of cancer over the past decade (1). Cancer immunotherapies based on immune evasion mechanisms, which are represented by B7-H1/PD-1 pathway targeting (anti-PD therapy), have achieved higher objective response rates in patients with considerably fewer immune-related adverse events than immune enhancement, indicating that normalization cancer immunotherapy has come of age with the strenuous efforts expended to enhance clinical efficacy. The concept of immune normalization emphasizes the importance of specifically correcting immune deficiencies to restore natural antitumor immune capacity (2). The B7 superfamily provides the second signal of the T-cell activation process, which is necessary to ensure an appropriate immune response; several members of the B7 superfamily, which is represented by B7-H1/PD-1, have been implicated in immune deficiency in the tumor microenvironment (TME) (3–5). The B7 superfamily can be divided into three groups in accordance with the signals that they transduct during T-cell activation: I) costimulatory, II) coinhibitory, and III) costimulatory/inhibitory (6).

B7 homolog 3 protein (B7-H3), also known as CD276, is an immune checkpoint molecule and a costimulatory/coinhibitory immunoregulatory protein that plays a dual role in the immune system (7). It was first cloned in 2001 from a cDNA library that was derived from human dendritic cells (DCs) (8). The human B7-H3 gene is located on chromosome 15, and the murine B7-H3 gene has been mapped to chromosome 9 (3). The human B7-H3 protein exists either as a transmembrane or soluble isoform. Transmembrane B7-H3 is a type I transmembrane protein that contains 316 amino acids and has a molecular weight of ~45–66 kDa (8, 9). It is composed of an extracellular domain, a transmembrane domain, and a short intracellular domain. The extracellular domain in murine B7-H3 (2IgB7-H3, B7-H3 VC) is composed of a single pair of immunoglobulin variable domain and constant domain and human B7-H3 (4IgB7-H3, B7-H3 VCVC) is composed of two pairs due to exon duplication (10, 11). Soluble B7-H3 (sB7-H3), which is cleaved from the surface by a matrix metallopeptidase (MMP) or produced through the alternative splicing of the intron, has also been detected in human sera (12, 13). The B7-H3 protein has also been found in the secretome, including exosomes and other extracellular vesicles (14). Asuthkar et al. found that B7-H3 induces greater exosome secretion and stimulates increased exosome size in D283 medulloblastoma cells (15).

TREM-like transcript 2 (TLT-2) has been identified as a potential receptor of B7-H3 (16). However, TLT-2 may not be the only receptor of B7-H3 considering that B7-H3 has many contradictory roles. In contrast to other immune checkpoints, B7-H3 not only influences innate and adaptive immunity but also regulates the aggressiveness of cancer cells through various nonimmunological pathways (17). Various anti-B7-H3 approaches have been studied in preclinical and clinical trials and have demonstrated their feasibility for clinical application (18).

## B7-H3 Is Highly Expressed in Different Types of Human Cancers

In most normal human tissues, B7-H3 mRNA is expressed widely, whereas the B7-H3 protein is relatively rarely present; the difference between the mRNA and protein expression patterns of B7-H3 suggests that B7-H3 has a tight post-transcriptional regulation mechanism (19). Evidence suggests that miR-124 may cause translational repression by playing a tumor suppressor role and targeting the 3ʹ-UTR of B7-H3 (20). Furthermore, miR-29 overexpression can inhibit B7-H3 expression levels, which play a crucial role in promoting medulloblastoma angiogenesis (21). Besides, B7-H3 expression is negatively regulated by miR-128 in colorectal cancer (CRC) (22).

**Figure 1:**
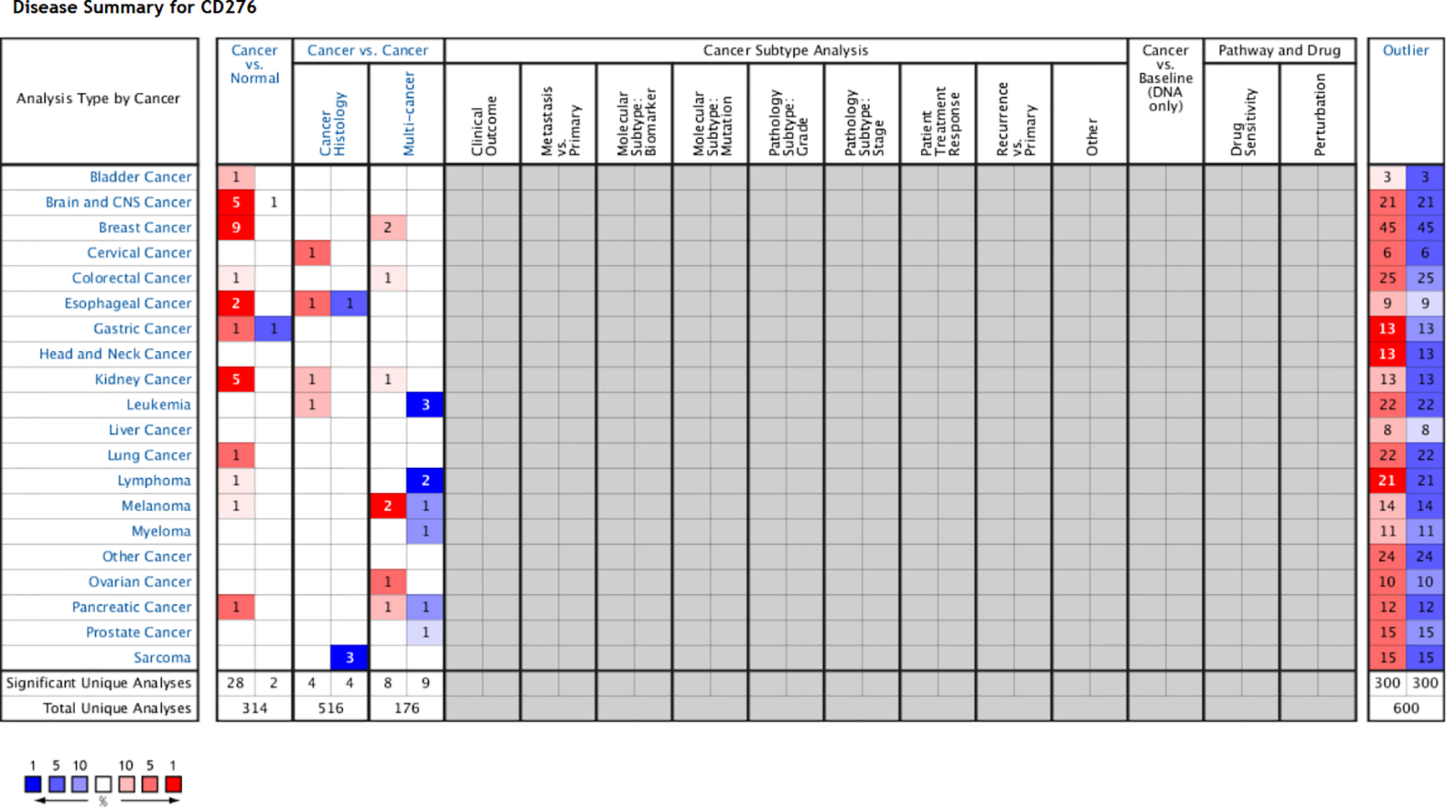
B7-H3 differential expression datasets in distinct cancers. P-value equals 0.05, fold change equals 2, red indicates high expression, and the darker the color, the higher the expression, blue indicates vice versa (only comparisons within the same row). Gray and blank means no data. Cell color is determined by the best gene rank percentile for the analyses within the cell (Note: an analysis may be counted in more than one cancer type). www.oncomine.org.

**Figure 2:**
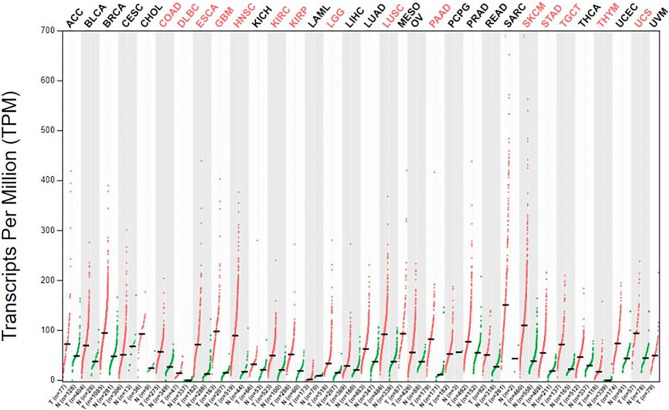
The gene expression profile across all tumor samples and paired normal tissue (dot plot). Labels upward the figure show different types of tumor marked different color, red show statistic data have significant difference, black show statistic data have no significant difference; The X axis is the number of tumor samples (T) in red and normal samples (N) in green for each tumor. The Y axis is transcripts per million (TPM). gepia.cancer-pku.cn

We obtained datasets on the differential expression of B7-H3 in distinct cancers from the Oncomine online database (**Figure 1**) and B7-H3 transcripts across all tumor samples and paired normal tissues from the Gene Expression Profiling Interactive Analysis (GEPIA) online database (**Figure 2**). We could find that CD 276 is highly expressed in both mRNA and protein level in tumor cells.

B7-H3 has been extensively studied in various cancers, including but not limited to breast cancer, lung cancer, ovarian cancer, brain tumor, gastric cancer, and squamous cell carcinoma (**Table 1**). Its presence has been correlated with worsened prognosis, poor survival, and recurrence rate. B7-H3’s capability to confer enhanced invasive and migratory properties has been further studied by using *in vitro* cancer models and is highlighted below.

**Table 1:** Expression and diverse roles of B7-H3 in multiple types of human cancers.

Cancer type	Case number	Positive rate (%)	Cell category	Function	Reference
**Breast cancer**	74	56.8 (42/74)	Cancer tissue	B7-H3 participated in the occurrence and metastasis of breast cancer	(23)
	74	43.2 (32/74)	Adjacent tissue		
**Non-small cell lung cancer**	82	74	Tumor samples	B7-H3 impaired anti-PD-1 therapy in NSCLC	(24)
**Ovarian cancer**	103	93	Tumor samples	B7-H3 downregulated T cell mediated antitumor immunity	(25)
**Meningioma**	21	76.2	Tumor cells	B7-H3 expression was elevated in patients with gene mutations related to the PI3K/AKT/mTOR pathway	(26)
	8	75	Tumor tissues	B7-H3 protein might play important roles in meningioma immune responses	(27)
**Gastric cancer**	120	69.2	Cancer tissues	B7-H3 silencing downregulates CXCR4	(28)
**Esophageal squamous cell carcinoma**	66	69.7	Cancer tissues	Knockdown of B7-H3 on tumor cells suppressed ESCC cell migration and invasion	(29)
**Cutaneous squamous cell carcinoma**	66	85	Tumor tissues	B7-H3 expression was the only parameter in immunocompetent individuals that was significantly different from that in immunosuppressed patients	(30)

Not all clinical studies were included in this table due to the space limitation.

### B7-H3 in Breast Cancers

Breast cancer is the most frequently diagnosed cancer in women worldwide and the second leading cause of cancer deaths among women (31, 32). A study on American Joint Committee on Cancer stages I to III primary breast cancers and normal breast specimens found that B7-H3 was expressed in 32 out of 82 primary breast tumors and compared with normal breast tissue, B7-H3 expression in primary tumors had a significant correlation with increased tumor size and lymph vascular invasion (33). By utilizing immunohistochemistry (IHC), Yu et al. discovered that the positive rate of B7-H3 was 56.8% (42/74) in 74 specimens of breast cancer tissues and was higher than 43.2% (32/74) in 74 specimens of adjacent tissues (23).

### B7-H3 in Lung Cancers

B7-H3 has been studied in non-small cell lung cancer (NSCLC). NSCLC is one of the cancers with the highest morbidity and mortality worldwide (34). Studies have shown that B7-H3 molecules are closely related to the invasion, metastasis, proliferation, and prognosis of NSCLC tumors (35). Yonesaka et al. evaluated B7-H3 expression levels in NSCLC tumors by using IHC and found that 74% of the tumor samples expressed B7-H3 with a staining pattern of 1+, 2+, or 3+ (24). Moreover, Wang et al. found that CD276 silencing inhibited cell invasion and migration by reducing integrin-associated protein expression (35). These studies indicated that in NSCLC, the presence of B7-H3 contributes to the capability of malignant neoplasms to progress and metastasize.

### B7-H3 in Ovarian Cancers

B7-H3 has also attracted interest in the field of ovarian cancer research. By applying IHC, Zang et al. found that B7-H3 expression was present in 96 out of 103 (93%) ovarian tumors (25). Notably, in the ovarian TME, stromal cells express B7-H3 at higher levels than tumor cells (36). Cai et al. assessed the expression of B7 checkpoint molecules in OvCa and found that B7-H3, but not PD-L1, was highly expressed and that the high expression of B7-H3 was associated with dysfunction in tumor-infiltrating T cells (37).

### B7-H3 in Brain Cancers

Multiple studies have shown that B7-H3 is present in a range of brain cancers. Different types of gliomas have different B7-H3 expression levels. Weak B7-H3 expression was found in oligodendroglioma and choroid plexus papilloma specimens. B7-H3 was expressed at moderate-to-high levels in medulloblastoma, ependymocytoma, glioblastoma, anaplastic astrocytoma, glioblastoma multiforme, and diffuse intrinsic pontine glioma (3, 38). The differential expression of B7-H3 in different types of gliomas requires further study. A previous study showed that all 16/21 meningioma specimens presented high B7-H3 expression with strong membrane staining in almost 100% of tumor cells and that B7-H3 expression in the five remaining meningioma tissues was moderate to high (26). B7-H3 expression was detected in 75%–90% of the tumor tissues in six out of eight cases, and extremely low levels of B7-H3 were detected in normal brain tissues (27). Wang et al. revealed that 2IgB7-H3, but not 4IgB7-H3, was specifically expressed in gliomas; they also demonstrated for the first time that 2IgB7-H3 was a valuable biomarker for the diagnosis of glioma (39). Proctor et al. found that B7-H3 was the most prevalent and abundant inhibitory immune checkpoint protein quantified in meningioma (26).

### B7-H3 in Other Cancers

Li et al. demonstrated that B7-H3 promoted gastric cancer cell migration and invasion and that its upregulation enhanced tumor infiltration depth (28). Wang et al. found that B7-H3 was involved in the progression of esophageal squamous cell carcinoma and the tumor escape of immunosurveillance (29). Varki et al. discovered that in patients who were positive for HIV and had cutaneous squamous cell carcinoma, the significantly higher B7-H3 expression levels of tumor cells in immunocompetent patients than in immunosuppressed individuals was largely driven by reduced B7-H3 expression (30). Besides, Tetzlaff et al. found that B7-H3 expression in Merkel cell carcinoma (MCC)-associated endothelial cells correlates with locally aggressive primary tumor features and increased vascular density (40).

### B7-H3 in Normal Cells

Human B7-H3 protein is not expressed constitutively on monocytes, B, T, or NK cells but can be induced on these cell types (8, 11). Phorbol myristate acetate plus ionomycin can induce the surface expression of B7-H3 on T, B, and NK cells (11). Anti-IgM also promotes B7-H3 expression on murine B cells. Anti-CD40 can induce B7-H3 expression on murine macrophages and B cells. LPS stimulates B7-H3 protein expression on murine DC and macrophages. In murine DCs, B7-H3 mRNA expression is stimulated by IFN-γ but is suppressed by IL-4 (41). B7-H3 expression can be induced by the granulocyte–macrophage-colony-stimulating factor LPS on monocytes and by IFN-γ on DCs (8, 11). B7-H3 also has functions in somatic cells. B7-H3 is highly expressed on osteoblasts during embryogenesis and is crucial for osteoblastic differentiation and bone mineralization (42).

B7-H3 is overexpressed in numerous tumor types. It is not expressed or expressed at low levels in either lymphoid cells or lymphocytes but exhibits increased expression when induced. These expression patterns imply that B7-H3 may play an important role in tumor development and cancer immunity.

## The Signaling Pathways Mediated by B7-H3 in a Distinct Manner

The high expression of B7-H3 in tumor tissues has aroused researchers’ interest in the role of B7-H3 in the TME. A large body of experimental evidence indicates that B7-H3 can affect the progression of tumors through immune-dependent and nonimmune pathways.

### Immune-Dependent Direction

#### Co-Stimulatory Role

B7-H3 has been suggested to play conflicting molecular roles in the immune system. B7-H3 was originally identified as a co-stimulatory molecule. In the presence of the anti-CD3 antibody, human B7-H3 protein increases the proliferation of CD4+ and CD8+ T-cells and enhances cytotoxic T-cell activity (8). CRC-bearing mouse models treated with adenoviral B7-H3 showed suppressed tumor growth and reduced secondary metastasis occurrence with significantly higher frequencies of IFN-γ-producing CD8+ T cells and higher IL-12 levels than the control group mice (43, 44).

#### Co-Inhibitory Role

**Figure 3:**
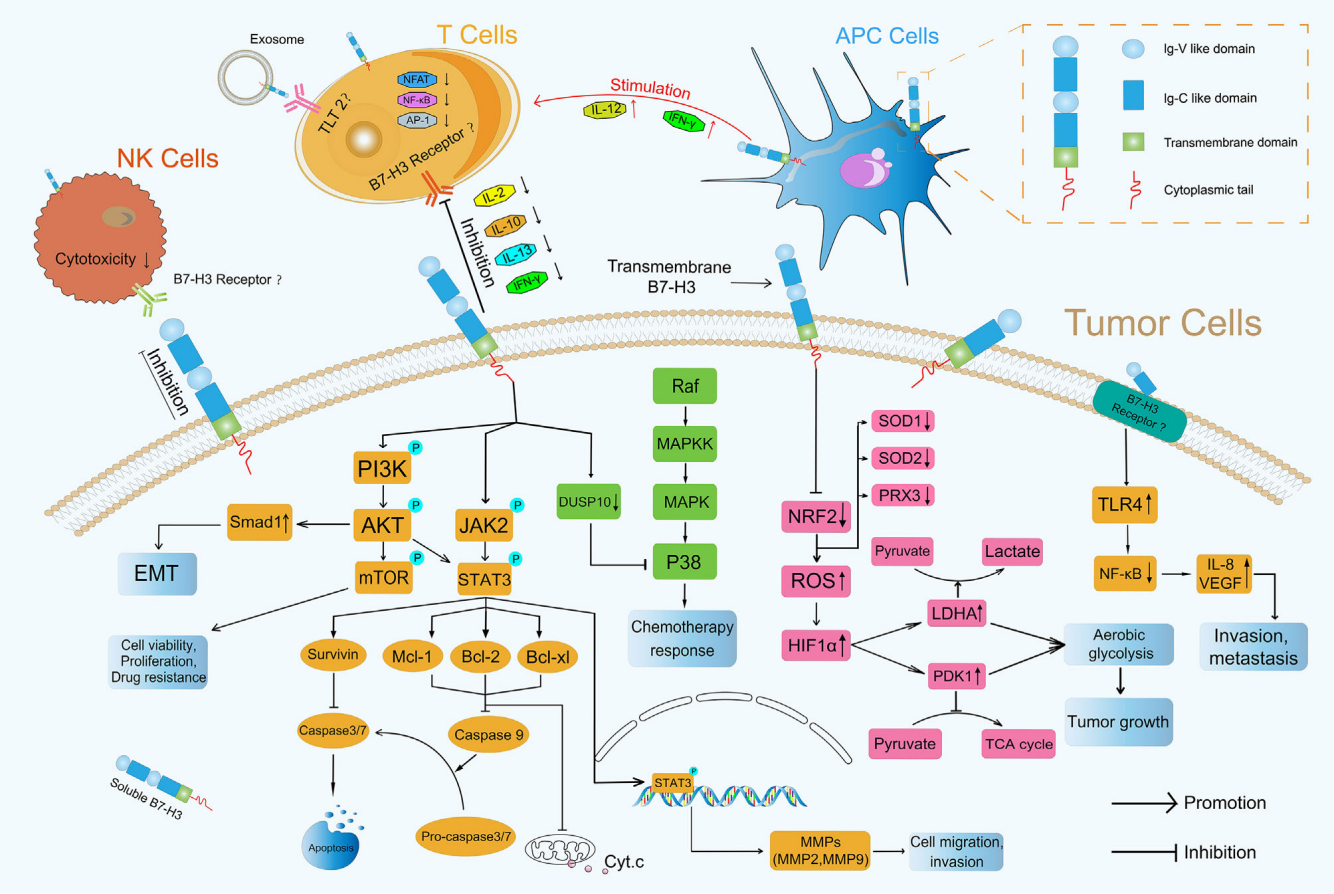
Roles of B7-H3 in TME. B7-H3 can affect the progression of tumors through immune-dependent and nonimmune pathways.

In addition to its co-stimulatory role, B7-H3 plays a co-inhibitory role in antitumor immunity. Several current studies have shown that B7-H3 inhibits the proliferation of CD4+ and CD8+ T-cells and reduces the production of IL-2 and IFN-γ possibly through the suppression of NF-κB, the nuclear factor of activated T-cells, and activator protein-1-mediated signaling pathways (41, 45) (**Figure 3**). During T-cell activation, B7-H3 potently and consistently inhibits of T-cell proliferation and IFN-γ, IL-13, IL-10, and IL-2 production (**Figure 3**). In addition to its inhibitory effect on T-cells, B7-H3 inhibits NK cell activity. 4Ig-B7-H3-transfected CHO-K cells avoided NK-cell-mediated cytotoxicity with an unclear receptor (11, 46). Recently, Wang et al. found that cancer stem cells (CSCs) utilize B7-H3 to evade immune surveillance during head and neck squamous cell carcinoma initiation, development, and metastasis (47). The suppressive immune microenvironment shaped by B7-H3 helps cancer avoid immune destruction (48).

### Non-Immune Direction

In addition to its role in immunological pathways, B7-H3 has nonimmunological protumorigenic functions, such as the promotion of migration and invasion, antiapoptosis, cell viability, chemoresistance, and endothelial-to-mesenchymal (EMT) transition. In tumor cells, it also participates in reprogramming metabolism through vital intracellular signal transduction pathways.

#### PI3K/AKT

Many articles have reported that the PI3K/AKT signaling pathway which is activated by phosphorylation is involved in the invasion of cancer cells (49–51) and participates directing immune cell differentiation and function (52). Li et al. found that B7-H3 overexpression promoted the migration and invasion of human bladder cancer cells and that B7-H3 knockdown suppressed the expression of MMP2 and MMP9 *via* the PI3K/AKT/STAT3 signaling pathway (53) (**Figure 3**). Nunes-Xavier et al. used API-2 (triciribidine, an AKT inhibitor) and RAD-001 (everolimus, a mTOR inhibitor) to target the PI3K/AKT/mTOR pathway and discovered that the inhibition of cell viability and proliferation in B7-H3 knockdown tumor cells was enhanced relative to that in their counterparts (54) (**Figure 3**). Jiang et al. found that B7-H3 upregulated Smad1 expression *via* the PI3K/AKT pathway, downregulated β-catenin and E-cadherin expression, and increased vimentin and N-cadherin expression, indicating that B7-H3 promoted EMT in colorectal cancer (55) (**Figure 3**). The expression of MMP2, MMP9 and EMT formation can contribute to mechanical microenvironment shaping in TME (48).

#### NF-κB

NF-κB transcription factors are activated as a response to a variety of signals (56). Wang et al. revealed that B7-H3 knockdown obviously reduced the phosphorylation levels of AKT, NF-κB, and STAT3 in HCT116 and RKO cells and that the NF-κB pathway had a major effect on B7-H3-induced VEGFA expression in CRC cells (57). Xie et al. proved that sB7-H3 first upregulated TLR4 expression, then activated NF-κB signaling, and finally promoted IL-8 and VEGF expression and demonstrated for the first time that sB7-H3 promoted the invasion and metastasis of pancreatic carcinoma cells through the TLR4/NF-κB pathway (58) (**Figure 3**).

#### Ras/Raf/MEK/MAPK

MAPK pathways regulate various cellular processes through four major pathways as defined by their MAPK effector: ERK1/2, ERK5, JNKs, and p38 MAPK (59). Flem-Karlsen et al. found that the knockdown of B7-H3 increased the *in vitro* and vivo sensitivity of melanoma cells to the chemotherapeutic agents dacarbazine and cisplatin in parallel with a reduction in p38 MAPK phosphorylation; they also observed the increased expression of dual-specific MAP kinase phosphatase (MKP) DUSP10 (a MKP known to dephosphorylate and inactivate p38 MAPK) in B7-H3 knockdown cells, indicating that B7-H3-mediated chemoresistance in melanoma cells is driven through a mechanism involving the DUSP10-mediated inactivation of p38 MAPK (60) (**Figure 3**).

#### JAK2/STAT3

The JAK/STAT signaling pathway is a critical controller of cellular survival and proliferation and is involved in cell antiapoptosis (61). The JAK2/STAT3 pathway activates some apoptosis suppressors, including survivin, Mcl-1, Bcl-xL, and Bcl-2, that block caspase cascades and apoptosis initiation in tumor cells (62). The direct inhibition of effector caspases 3 and 7 by survivin results in the suppression of apoptosis (63). Mcl-1, Bcl-2, and Bcl-xL inhibit the release of Cytochrome c (Cyt.c), thus preventing Cyt.c from reaching the threshold necessary for caspase cascades (64) (**Figure 3**). Several studies have demonstrated that B7-H3 performs an antiapoptotic role in tumorigenesis *via* the JAK2/STAT3 pathway. Liu et al. discovered that the knockdown of B7-H3 abrogated the phosphorylation of STAT3 through the inactivation of JAK2 and led to the downregulation of the direct target genes of STAT3 and to the reduction in survivin. By contrast, the overexpression of B7-H3 increased the phosphorylation of JAK2 and STAT3, indicating that the JAK2/STAT3 pathway contributes to B7-H3-mediated drug resistance (65) (**Figure 3**). Li et al. found that shRNA-mediated B7-H3 silencing inhibited AKT, ERK, and JAK2/STAT3 phosphorylation in the N87 gastric cancer cell line (28). Zhang et al. demonstrated that the overexpression of B7-H3 induced resistance to apoptosis in colorectal cancer cell lines by upregulating the JAK2-STAT3 signaling pathway; this effect thus potentially provides new approaches to the treatment of colorectal cancer (66). Recently, Lu et al. showed that B7-H3-mediated colon cancer cell resistance to the cytotoxicity of Vδ2 T cells involved a molecular pathway comprising STAT3 activation and decreased ULBP2 expression (67). However, how B7-H3 activates the downstream JAK2/STAT3 pathway remains unknown, and its underlying mechanism remains a point of conjecture (68). Other novel mechanisms that remain undiscovered must be explored in future investigations.

#### Glucose Metabolic Signaling Pathway

B7-H3 also plays a crucial role in glucose metabolic reprogramming. Cancer cell metabolism is characterized by an increase in glycolysis and lactate production even in the presence of abundant oxygen; this phenomenon is known as the Warburg effect or aerobic glycolysis (69). Aerobic glycolysis confers a growth advantage to cancer cells by providing energy and biosynthetic building blocks (70). Lim et al. demonstrated that B7-H3 regulated glucose metabolism through ROS-mediated HIF1a stabilization, which contributed to B7-H3-enhanced tumor growth; B7-H3 suppresses NRF2 transcriptional activity, which in turn reduces transcription of the antioxidant enzymes SOD1, SOD2, and PRX3; B7-H3-induced ROS then stabilized HIF1α, thus increasing the expression of the glycolytic enzymes LDHA and PDK1, an effect that promoted pyruvate conversion into lactate while inhibiting pyruvate flux through the TCA cycle (71) (**Figure 3**), contributing to tumor metabolism microenvironment shaping (48). Moreover, in CRC cells, B7-H3 mediated the activation of STAT3 and the subsequent expression of HK2 to promote glycolysis (72).

## B7-H3-Based Tumor Immunotherapy Strategies

Immunotherapy is a novel individualized treatment strategy wherein the immune system is activated or suppressed to amplify or diminish an immune response. It has been developed rapidly for the treatment of various forms of cancer in recent years. Immune-based therapies are gaining attention due to improvements in their clinical outcomes. The abnormal expression of B7-H3 is a possible biomarker and a promising immune checkpoint target for multiple cancer immunotherapy approaches, especially its expression on cancer initiating cells (CICs), since their eradication is a requirement for an anti-tumor therapy to be effective (73). Recent advances in molecular biology and antibody engineering have enabled targeting B7-H3 on the basis of multiple mechanisms. Information about clinical trials can be seen in **Table 2**.

**Table 2:** Summary of the clinical trials on anti B7-H3 antibodies for hematologic and solid tumor malignancies.

Trial number	Description	Drug	Trial stage	Start date	Completion date	Status
**Targeting B7-H3 through ADCC**						
NCT01391143	Refractory cancer, melanoma, prostate, solid tumors	Enoblituzumab (MGA271)	Phase I	July, 2011	April 18, 2019	Completed
NCT02982941	Pediatric patients with relapsed or refractory solid tumors	MGA271	Phase I	December, 2016	May 22, 2019	Completed
NCT02923180	Localized intermediate- and high-risk prostate cancer	MGA271	Phase II	October, 2016	October, 2021	Active but not recruiting
**Targeting B7-H3 through ADC therapies**						
NCT03729596	Advanced solid tumors	MGC018 with or without MGA012	Phase I/II	November 21, 2018	May, 2025	Recruiting
NCT02475213	Patients with melanoma, squamous cell cancer of the head and neck, NSCLC, and other cancers	MGA271 with pembrolizumab	Phase I	July, 2015	October, 2022	Recruiting
**Targeting B7-H3 with bispecific antibodies**						
NCT02628535	Patients with unresectable or metastatic neoplasms	Orlotamab (MGD009)	Phase I	September, 2015	November 25, 2019	Terminated
**Targeting B7-H3 with CAR T cells**						
NCT04185038	Diffuse Intrinsic Pontine Glioma/Diffuse Midline Glioma and Recurrent or Refractory Pediatric Central Nervous System Tumors	–	Phase I	December, 2019	May, 2041	Recruiting
NCT04077866	Patients with Recurrent or Refractory Glioblastoma	–	Phase I/Phase II	May 1, 2022	July 1, 2024	Recruiting
NCT04385173	Patients with Recurrent and Refractory Glioblastoma	–	Phase I	June 1, 2020	July 1, 2022	Recruiting
NCT04483778	Recurrent/Refractory Solid Tumors in Children and Young Adults	–	Phase I	July 13, 2020	December, 2040	Recruiting
**Synergistic options with anti–B7-H3 therapies**						
NCT02381314	Patients with melanoma, NSCLC, and other cancers	MGA271, ipilimumab	Phase I	March 26, 2015	September 26, 2018	Completed
NCT04129320	Squamous Cell Carcinoma of the Head and Neck	MGA271, MGA012	Phase II/III	October, 2019	October, 2025	Not yet recruiting
NCT02475213	Safety Study in Refractory Cancer	MGA271, pembrolizumab or MGA012	Phase I	July, 2015	October, 2022	Active but not recruiting
NCT03406949	Relapsed/Refractory Cancer	MGD009/MGA012	Phase I	February 27, 2018	December, 2022	Active but not recruiting
NCT01099644	Patients with Desmoplastic Small Round Cell Tumors and Other Solid Tumors	^131^I-8H9	Phase I	April, 2010	September, 2020	Active but not recruiting
NCT04022213	Patients with Desmoplastic Small Round Cell Tumors and Other Solid Tumors	^131^I-8H9	Phase II	July 15, 2019	July 2024	Recruiting
NCT04167618	Recurrent or Refractory Medulloblastoma	^177^Lu-DTPA-8H9	Phase 1/Phase 2	January 15, 2021	December 15, 2024	Not yet recruiting
NCT04315246	Leptomeningeal Metastasis from Solid Tumors	^177^Lu-DTPA-8H9	Phase 1/Phase 2	December 31, 2020	December 31, 2024	Not yet recruiting

Not all clinical studies were included in this table due to the space limitation.

### Targeting B7-H3 With Blocking mAbs

Blocking mAbs can partially or completely neutralize inhibitory ligand-to-receptor interactions, thus allowing effector functions (18). The use of blocking mAbs against the immune checkpoints CTLA-4, programmed cell death protein 1 (PD-1), and PD-1 ligand 1 (PD-L1) has demonstrated significant clinical success in patients with a variety of cancers (74–76). This successful experience can be applied to B7-H3 as well. B7-H3 blocking with mAbs has been shown to increase CD8+ T-cell and NK-cell tumor infiltration, reduce tumor growth, and prolong survival in mouse models of hematopoietic cancers, ovarian cancer (37), melanoma (77) and CRC (78). However, the translation of this strategy into the clinical setting has been hampered by the lack of human B7-H3-specific blocking mAbs.

### Targeting B7-H3 Through ADCC

**Figure 4:**
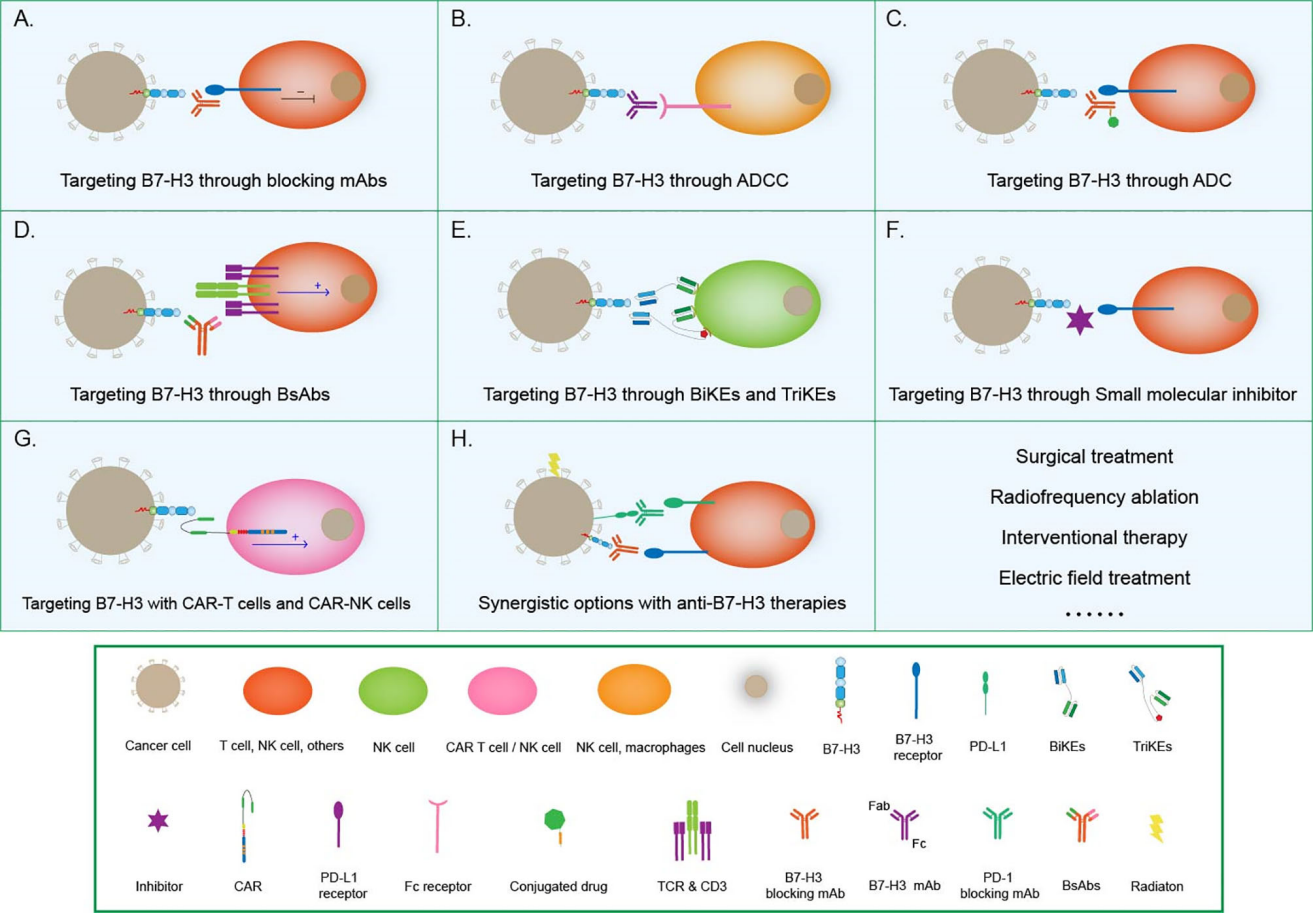
Tumor immunotherapy strategies based on B7-H3. **(A)** Targeting B7-H3 with blocking mAbs; **(B)** Targeting B7-H3 through ADCC; **(C)** Targeting B7-H3 through ADC therapies; **(D)** Targeting B7-H3 with CD3-engaging BsAbs; **(E)** BiKEs and TriKEs; **(F)** Targeting B7-H3 with small-molecule inhibitors; **(G)** Targeting B7-H3 with CAR T cells and CAR-NK cells; **(H)** Synergistic options with anti B7-H3 therapies.

Antibody-dependent cell-mediated cytotoxicity (ADCC) refers to the binding of the antibody Fab to malignant cells. Moreover, Fc can bind to FcR on the surfaces of killer cells to mediate the direct killing of target cells. Loo et al. developed MGA271, a B7-H3-reactive, Fc-engineered mAb that mediates potent antitumor activity *in vitro* and in tumor xenografts; this characteristic, together with the favorable safety profile of MGA271 in cynomolgus monkey toxicology studies, supports its exploration in the treatment of B7-H3-positive cancers (79) (**Figure 4**).

### Targeting B7-H3 Through Antibody–Drug Conjugates Therapies

Antibody–drug conjugates (ADCs) combine the target specificity of a mAb with cytotoxic agents to deliver the cytotoxic agents to a tumor and improve therapeutic indexes. Scribner et al. developed MGC018, an anti-B7-H3 ADC that incorporated an aduocarmycin-based DNA alkylating payload *via* a cleavable valine–citrulline linker. MGC018 exhibited potent antitumor activity in a range of human tumor xenografts, mediated bystander killing, and showed a favorable safety profile in cynomolgus monkeys (80).

### Targeting B7-H3 With CD3-Engaging Bispecific Antibodies

Bispecific antibodies (BsAbs) are another option that is beginning to pick up steam in the area of tumor immunotherapy. BsAbs are artificially generated antibodies that are composed of the fragments of two distinct Abs and combining two specificities. One arm can bind to the CD3 component of the TCR complex on T cells, whereas the other arm recognizes a tumor-specific antigen, such as B7-H3. In this way, T cells are recruited to the tumor site and activated to kill cancer cells (81). The anti-CD3 antibody that is chemically conjugated with the anti-B7-H3 mAb antibody has been clinically approved (82). The anti-CD3 × anti-B7-H3 bispecific antibody (B7-H3 Bi-Ab) was then used to direct activated T cells to kill tumor targets (83). The activated T cell armed with B7-H3 Bi-Ab exhibited increased specific cytotoxicity and cytokine production and suppressed B7-H3-positive cancer growth in the SCID–Beige murine model.

### Bi and Tri-Specific Killer Engagers

Bispecific killer engagers (BiKEs) or trispecific killer engagers (TriKEs) form an antigen-specific immunological synapse between NK and tumor cells, thereby triggering NK-cell-mediated tumor cell lysis. BiKEs consist of an anti-CD16 scFv linked to an scFv that is specific for a tumor-expressed antigen, and TriKEs comprise the two scFvs mentioned above and a cytokine, most frequently IL-15 (84). Vallera et al. generated a B7-H3/IL-15 TriKE that used the scFv of the B7-H3-specific mAb 376.96; when deployed against PDAC, this TriKE resulted in a significant reduction in tumor load *in vitro* and in murine models (85). The same group had bioengineered a second-generation TriKE with human IL-15 as a modified crosslinker between an anti-B7-H3 scFv and a humanized camelid anti-CD16 single domain antibody. The latter allowed the improved function of IL15, thus enhancing the activation and proliferation of NK cells and the killing of ovarian cancer cells *in vitro* and in murine models (86).

### Targeting B7-H3 With Small-Molecule Inhibitors

In addition to conventional therapeutic mAbs, small-molecule inhibitors have also begun to capture interest in the immune-oncology field (87). Small-molecule inhibitors are low-molecular-weight organic compounds (dinucleotides and peptides) that bind to specific biological targets, blocking specific antigen-antibody binding. They are readily used because of their advantages, including cheap manufacturing costs, ease of delivery due to their oral administration route, excellent tissue distribution due to their size, and short half-lives, over antibodies. A small-molecule inhibitor can be designed on the basis of the FG loop of the IgV domain of B7-H3 that is involved in T-cell activation to target this specific ligation area (88). Small molecule immunotherapy can provide an alternative treatment modality either alone or complementary to or synergistic with extracellular checkpoint mAbs to address low clinical response and drug resistance. The future research should continue to focus on discovery of novel small molecules with distinct chemo-types and higher potency (89).

### Targeting B7-H3 With Chimeric Antigen Receptor T Cells and Chimeric Antigen Receptor NK Cells

Chimeric antigen receptor (CAR) T-cell technology is another effective way to target B7-H3 for immunotherapy. Autologous T cells or NK cells are engineered with a CAR that targets a tumor antigen and adoptively transferred to patients to kill cancer cells. Thus far, this technology has been successfully applied only in hematologic cancers. Although this area of research is challenging, efforts are being made to translate CAR-T cell therapy into the treatment of solid tumors (18). B7-H3-redirected CAR-T cells can effectively control GBM growth (90, 91) and are highly active against atypical teratoma-like rhabdomyoma *in vitro* and in xenograft murine models (92). Majzner et al. reported a CAR-T cell system directed at B7-H3 with strong activity against a wide array of xenograft pediatric cancer models, including liquid, solid, and central nervous system (CNS) tumors; they also demonstrated that, as has emerged for many CAR therapeutics, CAR T cell activity is dependent on antigen density (38). Furthermore, Yang et al. developed a tandem CAR T cell system that exhibited enhanced antitumor activity and tumor control in several preclinical models (93). Tang et al. presented the results of the first-in-human clinical study on B7-H3-targeted CAR T cells for the treatment of recurrent anaplastic meningioma and provided evidence that the local delivery of B7-H3-targeted CAR T cells could suppress tumor progression without off-tumor toxicity or serious side effects, thus indicating the tolerability, safety, and potential efficacy of this therapy (94). Recently, NK-cells have been used to generate CAR-NK cells, which controlled the growth of human NSCLC cells grafted in murine models and prolonged survival (95). Lei et al. reported that a pan-histone deacetylase inhibitor can enhance the antitumor activity of B7-H3-specific CAR T cells in solid tumors (96).

### Synergistic Options With Anti–B7-H3 Therapies

With the successful experience of traditional immunotherapy, Combination therapy for improving the effect of immunotherapy and the survival rate of patients through combination of different immunotherapies has attracted increasing attention. Recent studies have shown that the combination of a variety of chemotherapeutics with checkpoint inhibitors exerts great synergistic effects that enhance the prospects of their full utilization in standard clinical practice.

Combination therapy involving multiple immune checkpoint inhibitors is emerging rapidly as a means for cancer treatment. Larkin et al. found that in patients with PD-L1-negative tumors, the combination of Nivolumab (PD-1 blockade) and ipilimumab (CTLA-4 blockade) was more effective than either agent alone (97). Xu et al. developed anti-B7-H3/PD-1 bispecific fusion proteins that simultaneously engaged the tumor-associated marker B7-H3 and the immune-suppressing ligand PD-L1 and enhanced ADCC to promote potent and highly selective tumor killing (98). Yonesaka et al. discovered that anti-B7-H3 immunotherapy combined with anti-PD-1/PD-L1 antibody therapy is a promising approach for the treatment of B7-H3-expressing NSCLCs (24).

B7-H3 gene silencing and combination drug therapy can also improve the rate of tumor elimination. Zhang et al. demonstrated that in U937 cells, B7-H3-targeting shRNA significantly enhanced sensitivity to chemotherapeutic drugs (99). Liu et al. examined the role of B7-H3 in paclitaxel resistance in several metastatic breast cancer cell lines; their results indicated that the B7-H3-shRNA-induced knockdown of the B7-H3 protein in these cells resulted in increased sensitivity to paclitaxel (65). Both studies showed that silencing B7-H3 significantly enhanced tumor cell chemosensitivity and drug-induced apoptosis, thus providing a rationale for the potential synergistic effects between the B7-H3 blockade and chemotherapy or targeted therapy for patients with a variety of cancers.

Radiation is an additional avenue that can be considered for application in a future clinical setting in combination with B7-H3 targeting. Twyman-Saint Victor et al. demonstrated that PD-L1 on melanoma cells allowed tumors to escape anti-CTLA4-based therapy, and the combination of radiation, anti-CTLA4, and anti-PD-L1 promoted response and immunity through distinct mechanisms (100). 8H9 is distinct from other B7-H3–specific antibodies in that it binds to the FG loop of B7-H3, a region that is critical to its immunologic function (101).

Given the multiple steps involved in anticancer immunity, the potential to enhance cancer immunotherapy *via* rational combinations by modulating different biological steps in immunity simultaneously or in rapid sequence is quite broad (102). Besides immunotherapies, some non-immunotherapies such as surgical treatment (103), radiofrequency ablation (104), interventional therapy (105), electric field treatment (106) should been also taken into consideration.

## Conclusions and Outlook

B7-H3 is a novel immune checkpoint from the B7 family. In this review, we analyzed the transcription and expression levels of B7-H3 in different tumors by utilizing bioinformatics tools, provided a comprehensive view of B7-H3’s role in the TME, and summarized different B7-H3-based cancer immunotherapy strategies along with their corresponding clinical trials. The prospect of B7-H3 as a target for cancer immunotherapy which stems from its special expression patterns on tumor cells and the safety profile has stimulated the progress of B7-H3-targeting therapeutic strategies. The success of immunotherapies such as targeting PD-1 and CTLA4, also provide researchers’ example and direction to develop new immunotherapy that target B7-H3. However, the unknown identity of the B7-H3 receptor greatly hinders the development of B7-H3 antagonists. Improving the understanding of B7-H3-mediated molecular processes for the regulation of tumorigenesis will open new avenues for developing novel therapeutic strategies for human cancers. Notably, organoids have attracted increasing attention in tumor research in recent years given their advantageous capability to reproduce tissue structure and organ function. The future trend of tumor immunotherapy involves studying cell therapy in different organoids on the basis of the new immune checkpoint B7-H3. To develop the diagnostic and therapeutic potential of B7-H3 completely, its expression in serum, pre-malignant lesions, tumor-associated vasculature, CSC, CIC, metastases and recurrence requires further investigation. Future studies aiming to delineate the precise cellular and molecular mechanisms based on B7-H3-mediated tumor promotion will provide further insights into the cell biology of tumor development and cancer immunotherapy.

## Author Contributions

The corresponding author W-LJ instructed the manuscript completion. The first author W-TZ contributed to manuscript writing and revising. All authors contributed to the article and approved the submitted version.

## Funding

This study was supported by grants from the National Key Research and Development Program of China (no. 2017FYA0205302) to W-LJ.

## Conflict of Interest

The authors declare that the research was conducted in the absence of any commercial or financial relationships that could be construed as a potential conflict of interest.

## Abbreviations

PD-1, Programmed cell death protein 1; TME, Tumor microenvironment; B7-H3, B7 homolog 3 protein; DCs, Dendritic cells; MMP, Matrix metallopeptidase; TLT-2, TREM-like transcript 2; CRC, Colorectal cancer; IHC, Immunohistochemistry; NSCLC, Non-small cell lung cancer; MCC, Merkel cell carcinoma; GM-CSF, Granulocyte–macrophage colony stimulating Factor; CSC, Cancer stem cells; EMT, Endothelial-to-mesenchymal; MKP, MAP kinase phosphatase; Cyt.c, Cytochrome c; NF-κB, Nuclear factor kappa B; CICs, Cancer initiating cells; JK , Janus kinase; STAT, Signal transducer and activator of transcription; NK, Natural killer cells; ADCC, Antibody-dependent cell-mediated cytotoxicity, ADCs, Antibody–drug conjugates; DCs, Dendritic cells; BsAbs, Bispecific antibodies; TriKEs, Trispecific Killer Engagers, CAR, Chimeric antigen receptor; CNS, Central nervous system.
